# Postoperative Analgesic Effect of Bilateral Quadratus Lumborum Block (QLB) for Canine Laparoscopic Ovariectomy: Comparison of Two Concentrations of Ropivacaine

**DOI:** 10.3390/ani13233604

**Published:** 2023-11-22

**Authors:** Massimiliano Degani, Chiara Di Franco, Hamaseh Tayari, Aida Fages Carcéles, Giacomo Figà Talamanca, Charlotte Sandersen, Angela Briganti

**Affiliations:** 1 Department of Veterinary Sciences, Veterinary Teaching Hospital “Mario Modenato”, University of Pisa, 56122 Pisa, Italy; chiara.difranco@phd.unipi.it (C.D.F.); aida.fages92@gmail.com (A.F.C.); g.ftalamanca@gmail.com (G.F.T.); angela.briganti@unipi.it (A.B.); 2 Clinical Department for Companion Animal Sciences, Faculty of Veterinary Medicine, University of Liège, 4000 Liège, Belgium; charlotte.sandersen@uliege.be; 3 Southern Counties Veterinary Specialists (SCVS), Forest Corner Farm, Hangersley, Ringwood, Hampshire BH24 3JW, UK; hamasehtayari@gmail.com

**Keywords:** analgesia, dog, quadratus lumborum block, laparoscopic ovariectomy, ultrasound-guided locoregional anesthesia

## Abstract

**Simple Summary:**

Laparoscopic ovariectomy is currently considered the gold standard for neutering female dogs thanks to the reduced surgical trauma, lower perioperative pain, and decreased hospitalization stays. Perioperative analgesic management becomes crucial to reduce the occurrence of uncontrolled post-surgery stress response. The quadratus lumborum block is an ultrasound-guided block capable of providing somatic and visceral analgesia to the abdomen. The aims of this study were (I) to evaluate the efficacy of the transversal quadratus lumborum block performed with two different concentrations of ropivacaine (0.5% and 0.33%) in prolonging the time to the first postoperative rescue analgesia in dogs undergoing laparoscopic ovariectomy, in comparison with a fentanyl-based protocol, and (II) to investigate the cardiovascular impact of the block on the arterial blood pressure. The results of this study showed a significantly longer time to the first postoperative rescue analgesia in groups receiving the block, in comparison with the group receiving a fentanyl-based protocol. Surprisingly, ropivacaine 0.33% produce a more important reduction in the arterial blood pressure than ropivacaine 0.5%. Further studies are needed to better assess the intraoperative efficacy of the quadratus lumborum block.

**Abstract:**

The aim of this study was to evaluate the effect of the transverse quadratus lumborum block (QLB_LQL-T_) on time to the first postoperative rescue analgesia in dogs submitted to laparoscopic ovariectomy. A total of twenty-three female dogs were included. Dogs were randomly assigned to receive a bilateral QLB_LQL-T_, performed either with 0.3 mL kg^−1^ ropivacaine 0.5% [group QLB_0.5%_ (*n* = 8)] or with ropivacaine 0.33% [group QLB_0.33%_ (*n* = 8)] or a fentanyl-based protocol [group No-QLB (*n* = 7)]. Dogs were premedicated intravenously (IV) with fentanyl 5 mcg kg^−1^, general anesthesia was induced IV with propofol and maintained with sevoflurane. Invasive mean arterial pressure (MAP) values were recorded five minutes before and five minutes after performing the QLB_LQL-T_. The short-form of the Glasgow composite measure pain scale was used every hour after extubation, and methadone 0.2 mg kg^−1^ was administered IV when pain score was ≥5/24. Kolmogorov–Smirnov test, ANOVA test combined with Tukey post hoc test, Student’s T-test and Chi-square test were used to analyze data; *p* < 0.05. Time from QLB_LQL-T_ to the first rescue analgesia was significantly longer in QLB_0.5%_ than in group QLB_0.33%_ and No-QLB. MAP pre- and post-block decreased significantly only in group QLB_0.33%_.

## 1. Introduction

Laparoscopic ovariectomy (LO) is currently considered the gold standard for neutering female dogs [1] thanks to the advantages over the traditional laparotomic technique, such as reduced surgical trauma, lower perioperative pain, and decreased hospitalization times [2,3,4]. In the absence of complications, dogs have been discharged between 8 and 12 h after surgery [5]. To permit such a prompt discharge, effective perioperative analgesia is essential to reduce the occurrence of uncontrolled post-surgery stress responses, including immunosuppression, overstimulation of the sympathetic nervous system, and anorexia [6]. Locoregional anesthesia (LRA) can provide a more stable anesthetic plane due to the sparing effect on inhalant anesthetic drugs and a reduction in the use of intra- and postoperative opioids [6,7,8]. 

Implementation of ultrasonography in modern LRA has led to a rapid growth in the use of so-called interfascial plane blocks (IFPBs) [9]. This class of blocks are relatively easy to perform safely and are considered as the minimally invasive evolution of locoregional anesthesia [10]. Among them, the ultrasound (US)- guided transversus abdominis plane (TAP) block aims to desensitize the thoracolumbar ventral rami of the spinal nerves (VRSN) and to provide somatic analgesia of the abdominal wall and peritoneum [11]: it has been proposed as part of a multimodal analgesic approach for dogs undergoing LO [12,13]. However, the US-guided quadratus lumborum block (QLB) has been shown to provide both somatic and visceral analgesia to the abdomen in both human and veterinary medicine [9,10,14], leading several authors prefer the QLB to the TAP block [15]. The target for the QLB is the fascia surrounding the quadratus lumborum (QL) muscle where the VRSN and the sympathetic trunk, responsible for the somatic and visceral innervation of the abdomen, lie. Garbin et al. described two lateral approaches in which the tip of the needle was positioned laterally to the QL muscle: a transversal (QLB_LQL-T_) and a longitudinal (QLB_LQL-L_) block, depending on the position of the US probe with respect to the spinal column [16,17]. Several other approaches have been evaluated in canine cadaveric studies [16,17,18,19,20,21]. Results showed a consistent spread of the injectate towards the VRSN between the first (L1) and fourth (L4) lumbar vertebrae, while spreading to the caudal thoracic vertebrae was less frequent. A variable spread of injectate solution along the sympathetic trunk between the thirteen thoracic (T13) and the third lumbar (L3) vertebrae has also been reported [16,17,18,19,20,21]. 

In human medicine, hypotension secondary to bilateral QLB has been reported in two case reports [22,23]. In veterinary medicine, transient respiratory arrest and retroperitoneal hematoma have been described as complications of bilateral QLB in dogs [24,25]. Currently, there is only one published case series regarding the analgesic efficacy of bilateral QLB in dogs undergoing ovariohysterectomy [14]. To the author’s knowledge, at the moment there are no randomized blinded studies in the veterinary literature regarding the use of QLB in dogs undergoing LO. 

This study aimed to investigate the effectiveness of the QLB_LQL-T_ performed using two different concentrations of ropivacaine (0.5% and 0.33%) at prolonging the time to the first postoperative rescue analgesia in dogs undergoing LO, and to compare these blocks with a systemic opioid-based protocol. We also investigated the impact of the QLB_LQL-T_ on the arterial blood pressure. We hypothesized that ropivacaine 0.5% would provide longer-lasting postoperative analgesic effect and produce a greater reduction in the arterial blood pressure versus ropivacaine 0.33%.

## 2. Materials and Methods

Twenty-four female dogs of different breeds, ages, and weights, undergoing LO at the Veterinary Teaching Hospital of the University of Pisa, were enrolled in the study. The Consolidated Standards of Reporting Trials (CONSORT) guidelines were applied [26]. Ethical approvals were obtained from the competent authorities (Nr. 25/2020). By signing the informed consent document, the owner agreed to enroll their dog in the study. Prior to anesthesia, each dog underwent a clinical examination, including body condition score (BCS) on a nine-point scale [27] and a complete hematological, biochemical, and coagulation screening. Based on the findings of the preoperative evaluation, only dogs with an American Society of Anesthesiologists (ASA) status 1, with a BCS ranging between 3 and 6 out of 9 were included in the study. Other exclusion criteria included problematic behavior, skin infection, or irritation at the site of the QLB_LQL-T_, and administration of any anti-inflammatory drugs within 24 h prior to surgery.

### 2.1. Preoperative Management

On the day of surgery, the following anesthetic protocol was applied to all dogs: after intravenous catheter insertion into one of the cephalic veins, a bolus of 5 mcg kg^−1^ of fentanyl (Fentadon, 50 mcg mL^−1^, Eurovet Animal Health B.V., Bladel, The Netherlands) was administered intravenously (IV) and lactated Ringer’s (RL) solution (Ringer Lattato, B. Braun S.p.a. Vet Care, Milan, Italy) at 5 mL kg^−1^ h^−1^ was initiated. After five minutes of pre-oxygenation by mask, general anesthesia was induced with propofol (Propovet multidose, 10 mg mL^−1^, Zoetis Italia S.r.l., Rome, Italy) IV to effect, until endotracheal intubation was feasible. The endotracheal tube was connected to a rebreathing system and anesthesia was maintained with sevoflurane (Sevoflo, Ecuphar S.r.l., Milan, Italy) in an oxygen–air mixture with a fraction of inspired oxygen (FIO_2_) of 0.6. A fraction of expired sevoflurane concentration (FE’Sevo) was initially set between 2 and 2.3%. Each dog was positioned in lateral recumbency. An arterial catheter was inserted at the level of the dorsal pedal artery and connected to a pre-calibrated transducer, zeroed to atmospheric pressure, positioned at the level of the right atrium and connected to a multi-parameter monitor (Mindray Beneview T5, Mindray Medical Italia S.r.l., Milan, Italy) for invasive blood pressure measurement. 

The hair in the abdomen and flank of each dog was clipped in the same way, to prevent recognition during the postoperative pain assessment. Dogs were randomly allocated into three groups: dogs in group QLB_0.5%_ received a bilateral QLB_LQL-T_ with 0.3 mL kg^−1^ of ropivacaine (Naropina, 10 mg mL^−1^, Aspen Pharma Trading Limited, Verona, Italy) 0.5% per side (total dose 3 mg kg^−1^); dogs in group QLB_0.33%_ received the same block with 0.3 mL kg^−1^ of ropivacaine 0.33% per side (total dose 2 mg kg^−1^); dogs in the control group (No-QLB) received a systemic fentanyl-based analgesic protocol and no LRA. The anesthetists in charge of intraoperative monitoring (C.D.F. or G.F.T.) were unaware of the concentration of ropivacaine used, though they were aware of the group No-QLB, for ethical reasons. Simple sequence randomization for 24 numbers divided into three columns was performed using the online website www.random.org (accessed on 1 July 2020). All blocks were performed by the same anesthetist (M.D.) using an along visual axis technique [28] with a dedicated veterinary ultrasound system (Sonosite S II Veterinary Ultrasound System, Fujifilm Italia S.p.a., Milan, Italy) and a linear ultrasound probe (HFL50, 15–6 MHz Linear Transducer). The QLB_LQL-T_ was performed as described by Garbin et al. [17] using an 85 mm echogenic needle for nerve blocks (Visioplex, Vygon Italia S.r.l., Padua, Italy). For each side (right and left), before performing the QLB_LQL-T_ and five minutes after infusion, invasive mean arterial pressure (MAP) values were recorded. A five minute interval was used after changing the recumbency prior to the MAP measurement and the next block. Time from the execution of the block to starting of surgery (T_B-S_) was also recorded.

### 2.2. Intraoperative Management

Subsequently, dogs were transported to the operating room and connected to an anesthetic workstation (Avance CS^2^, GE Healthcare, Bensalem, PA, USA). Volume-controlled ventilation was set to maintain end-expiratory CO_2_ (EtCO_2_) between 35 and 45 mmHg. During anesthesia, heart rate (HR), peripheral arterial hemoglobin saturation (SpO_2_), systolic, mean, and diastolic invasive arterial blood pressures (SAP, MAP, DAP), respiratory rate (fR), EtCO_2_, and FE’Sevo were continuously monitored and recorded every five minutes. All dogs were operated by the same surgeon, using the two-port LO technique, with transabdominal suspension suture for ovarian traction [2]. Sevoflurane was regulated to maintain a surgical anesthetic plane, defined as absence of palpebral reflex and voluntary movements, and a mild jaw tone [29]. 

HR and MAP registered five minutes before the start of the surgery were recorded as T0 and considered as baseline. A 20% increase in the cardiovascular variables from the corresponding values at T0 was considered as a sign of nociception [30] and a bolus of 2.5 µg kg^−1^ of fentanyl was administered IV. In case variables did not return to T0 within five minutes, fentanyl infusion was started at an increasing rate between 2 and 15 mcg kg^−1^ h^−1^ until return to basal values. The management of intraoperative nociception for dogs in group No-QLB was conducted as for any other procedure performed in our institution, in which LRA is not used; a bolus of 2.5 µg kg^−1^ of fentanyl was administered IV at T0, then a fentanyl infusion was started at a rate between 2 and 15 mcg kg^−1^ h^−1^ to maintain HR and MAP within 20% of baseline values during surgery [6]. In the QLB_0.5%_ and QLB_0.33%_ groups, the time of administration of fentanyl, if necessary, and the surgical step responsible for the nociception response were recorded. The following were considered surgical steps of LO: S_1_, placement of the drapes and Backhaus forceps; S_2_, skin incision; S_3_, introduction of the trocars and insufflation of the abdomen; S_4_, traction of the first ovary; S_5_, cauterization of the first ovary; S_6_, traction of the second ovary; S_7_, cauterization of the second ovary; S_8_, deflation of pneumoperitoneum and extraction of the trocars; S_9_, suturing of the skin. 

In case of hypotension (MAP < 60 mmHg), 5 mL kg^−1^ of Ringer lactate was administered IV over five minutes. If hypotension persisted, dopamine (Dopamina Hospira, 200 mg mL^−1^, Hospira Italia, Naples, Italy) infusion was started at 5 mcg kg^−1^ min^−1^ and increased by 0.5 mcg kg^−1^ min^−1^ every five minutes until the MAP was above 60 mmHg. In presence of bradycardia (HR < 60 bpm) and concomitant hypotension, atropine (Atropina Solfato 1 mg mL^−1^, A.T.I. Azienda terapeutica veterinaria S.r.l., Naples, Italy) at 20 mcg kg^−1^ was administered IV. At the end of surgery, administration of sevoflurane was interrupted and, when the dog returned to spontaneous ventilation, mechanical ventilation was stopped. Each dog was then moved to the recovery room and tracheal extubation was performed once the swallowing reflex returned. The duration of anesthesia and surgery and time from the block to starting of surgery were recorded.

### 2.3. Postoperative Management

One hour after extubation, an anesthetist (C.D.F. or G.F.T.), different from the one performing the block and the one monitoring the intraoperative phase and blinded to the treatment allocation, performed the pain assessment using the short-form Glasgow Composite Measure Pain Scale (SF-GCMPS) [31] every hour up to 8 h starting from the QLB_LQL-T_ execution. In case of a score ≥5/24, methadone (Semfortan, 10 mg mL^−1^, Dechra, Turin, Italy) 0.2 mg kg^−1^ IV and carprofen (Rimadyl, 50 mg mL^−1^, Zoetis Italia S.r.l., Rome, Italy) 2 mg kg^−1^ were administered IV, and the postoperative pain monitoring for the purposes of this study was interrupted. If the score was <5/24, up to 720 min of assessment, only carprofen 2 mg kg^−1^ IV was administered, and the study monitoring was stopped. The time from the block to the first postoperative methadone administration, if necessary, was recorded and compared between groups.

### 2.4. Statistical Analysis

The sample size calculation with an alpha of 0.05 and power of 80%, to detect a difference of 120 min in the time for the first postoperative methadone administration between group QLB_0.5%_ and group QLB_0.33%_. resulted in a minimum of seven dogs for each group. The calculation was done considering a mean duration of analgesia from ropivacaine 0.33% of about 550 ± 80 min [32]. We decided to enroll eight animals for each group, to face any eventual losses of dogs or higher standard deviation. These criteria gave a minimum number for each group of seven. Data were analysed for distribution with a Kolmogorov–Smirnov test. Data not normally distributed were described by the median and range, while those normally distributed were described by the mean and standard deviation. An analysis of variance (ANOVA) test with a Tukey post hoc test was used to compare duration of anesthesia, surgery, and time from the block to the first postoperative rescue methadone, amongst the three groups. For pre- and post-block MAP values in the two groups that received the QLB_LQL-T_, a Student’s T-test was used for paired data. For HR, fR, SAP, MAP, FE’Sevo values, an ANOVA test for repeated data was used to evaluate the trend inside each group. A Chi-square test was performed to evaluate fentanyl boluses in groups receiving the QLB_LQL-T_ and the use of vasoactive drugs in the three groups. *p* < 0.05.

## 3. Results

Twenty-three ASA I female dogs undergoing LO were included the study: one dog from group No-QLB was excluded because of conversion of the procedure into a laparotomic surgery due to intraoperative hemorrhage. Demographic data, T_B-S_, duration of anesthesia, and duration of surgery are summarized in Table 1. Median BCS was 4.5 (4–5) in all groups. 

MAP values five minutes before (78 ± 14 mmHg) and after (76 ± 11 mmHg) the block in group QLB_0.5%_ were not statistically different (*p* = 0.18), while a significant decrease (*p* = 0.002) in MAP five minutes after the block (from 76 ± 12 mmHg to 63 ± 8 mmHg) was recorded in group QLB_0.33%_.

**Figure 1:**
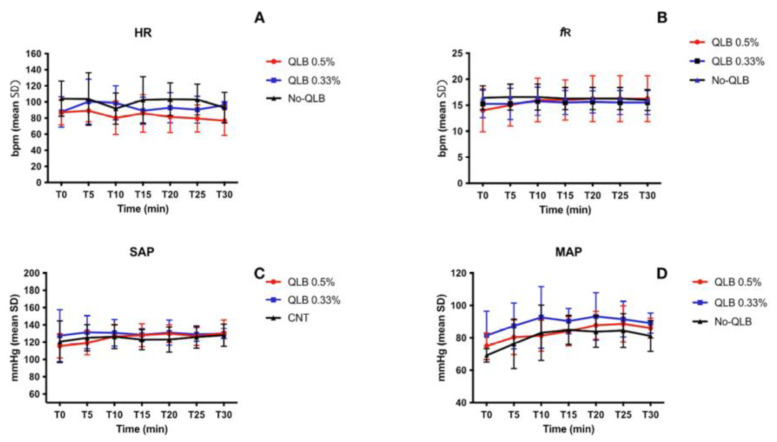
(**A**) mean values and standard deviation (SD) of intraoperative heart rate (HR) (beats per minute, bpm); (**B**–**D**) mean values and SD of respiratory rate (fR), systolic (SAP) and mean arterial pressure (MAP). QLB_0.5%_, quadratus lumborum block (QLB_LQL-T_) with ropivacaine 0.5%; QLB_0.33%_, quadratus lumborum block (QLB_LQL-T_) with ropivacaine 0.33%. No-QLB, systemic fentanyl-based analgesic protocol.

**Figure 2:**
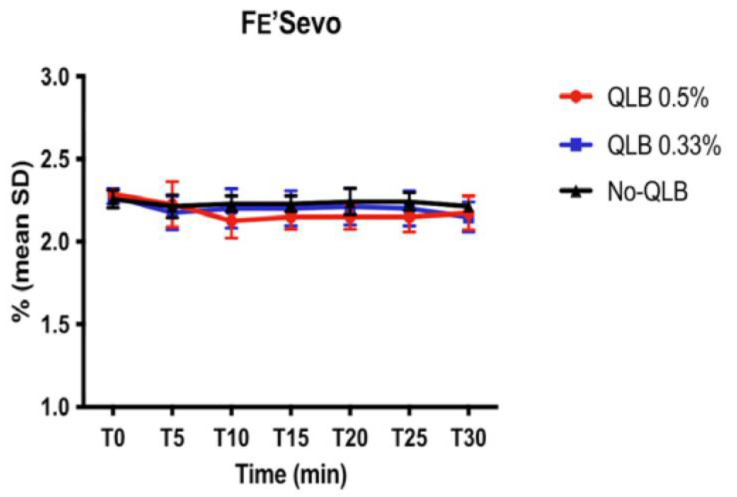
Mean values and standard deviation (SD) of fraction of expired sevoflurane (FE’Sevo). QLB_0.5%_, quadratus lumborum block (QLB_LQL-T_) with ropivacaine 0.5%; QLB_0.33%_, quadratus lumborum block (QLB_LQL-T_) with ropivacaine 0.33%. No-QLB, systemic fentanyl-based analgesic protocol.

Regarding the intraoperative values of HR, SAP, MAP, *f*_R_, FE’Sevo, no significant differences between T0 and the other intraoperative values for the three groups were detected (Figure 1A–D and Figure 2). 

One bolus of fentanyl was administered in 6/8 patients in group QLB_0.5%_ and in 7/8 patients in group QLB_0.33%_. No dogs in group QLB_0.5%_ and QLB_0.33%_ required fentanyl infusion. All dogs in group No-QLB received one bolus plus infusion of fentanyl (mean rate of 5.7 ± 3.2 mcg kg^−1^ h^−1^), to maintain the physiological variables within 20% of baseline values during surgery. Data regarding the distribution of intraoperative rescue fentanyl administration in group QLB_0.5%_ and QLB_0.33%_ are summarized in the Table 2. 

One dog in group QLB_0.5%_ and one in group QLB_0.33%_ required dopamine, while no one needed atropine; 4/7 dogs in group No-QLB received dopamine and 2/7 dogs received atropine. 

**Figure 3:**
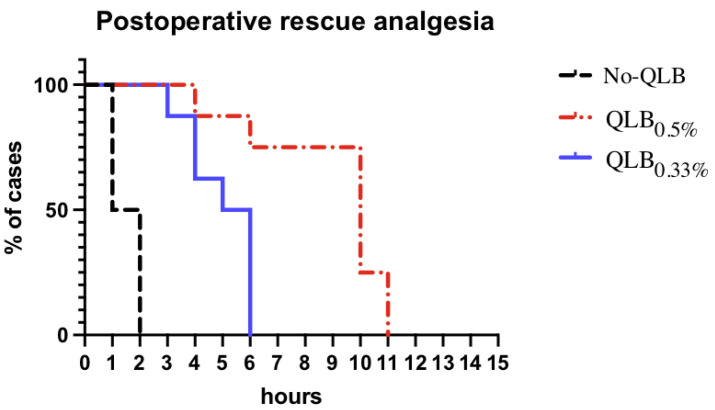
Kaplan–Meier analysis of time (hours) to first postoperative rescue analgesia required in a percentage (%) of cases in the three groups. QLB_0.5%_, quadratus lumborum block (QLB_LQL-T_) with ropivacaine 0.5%; QLB_0.33%_, quadratus lumborum block (QLB_LQL-T_) with ropivacaine 0.33%. No-QLB, systemic fentanyl-based analgesic protocol.

**Figure 4:**
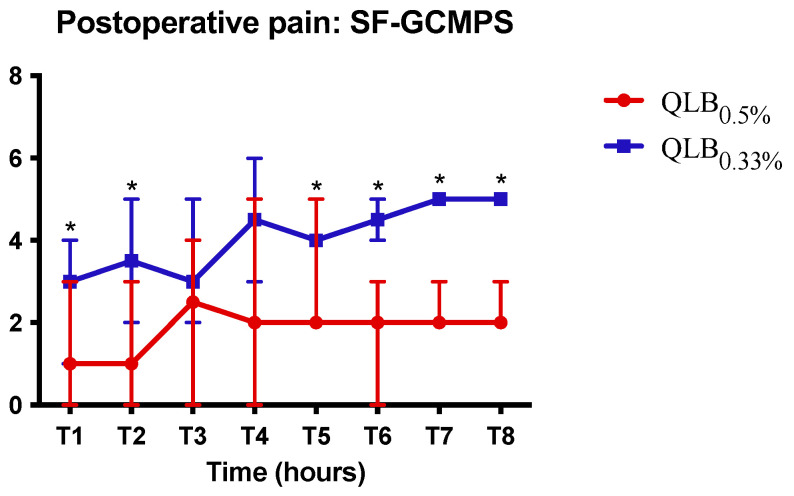
Median values and range of postoperative pain scores, short-form Glasgow Composite Measure Pain Scale (SF-GCMPS). QLB_0.5%_, quadratus lumborum block (QLB_LQL-T_) with ropivacaine 0.5%; QLB_0.33%_, quadratus lumborum block (QLB_LQL-T_) with ropivacaine 0.33%. No-QLB, systemic fentanyl-based analgesic protocol. * Significant difference between the two groups (*p* < 0.05).

Time from the block and the first postoperative methadone administration in group QLB_0.5%_, was 612 ± 164 min and was significantly longer than group QLB_0.33%_ (316 ± 113 min) (*p* = 0.002) and group No-QLB (59 ± 24 min) (*p* < 0.0001) (Figure 3); postoperative analgesia in the group QLB_0.33%_ was significantly longer than No-QLB (*p* = 0.0016). Postoperative pain scores were significantly different between the QLB groups at T1, T2, T5, T6, T7 and T8 (*p* = 0.007) with lower pain scores registered in group QLB_0.5%_ (Figure 4). No complications occurred in the postoperative period in any dog.

## 4. Discussion

The present study demonstrated that bilateral QLB_LQL-T_ with a volume of 0.3 mL kg^−1^ of ropivacaine 0.5% provides prolonged time until first postoperative rescue analgesia and reduced pain scores in female dogs undergoing LO, in comparison with dogs receiving the same block using ropivacaine 0.33% and dogs receiving a systemic fentanyl-based protocol. Surprisingly, results of this study indicated that ropivacaine 0.33% produced a more important reduction in the mean arterial blood pressure than ropivacaine 0.5%. 

In our study, QLB_LQL-T_ significantly prolonged the time to the first postoperative administration of methadone in comparison with group No-QLB. This finding is well documented in veterinary medicine, where the implementation of LRA led to a reduction in perioperative opioid requirements, improved quality of recovery, decreased morbidity, and hospitalization times [14,32,33,34]. Furthermore, the block performed with ropivacaine 0.5% delayed the first postoperative administration of methadone and reduced pain scores in comparison with ropivacaine 0.33%. These data support the hypothesis that the higher the concentration of LA, the higher the quality and duration of the block obtained, as already reported in the literature [32,33,35]. In a previous study, dogs receiving the QLB (0.4 mL kg^−1^ per side of bupivacaine 0.25%, 2 mg Kg^−1^), undergoing laparotomic ovariohysterectomy, were found to require the first post-operative methadone administration four hours after surgery [14]. This result does not differ much from ours. In our study, dogs in group QLB_0_._33_ (0.3 mL kg^−1^ per side of ropivacaine 0.33%, 2 mg Kg^−1^) and QLB_0.50%_ (0.3 mL kg^−1^ per side of ropivacaine 0.5%, 3 mg Kg^−1^) required rescue analgesia approximately 5 and 10 h after the execution of the block, respectively. Despite different surgeries, approaches and LAs were evaluated, it is possible to speculate that the delayed first postoperative methadone administration found in our study was produced by higher concentrations of ropivacaine used. 

The TAP block, in combination with intercostal blocks or alone, has been recently proposed as part of multimodal analgesic protocols in dogs undergoing LO [12,13]. Paolini et al. and Espadas-González et al. reported relatively no dogs required postoperative opioid administration up to 24 h post-surgery in both studies [12,13]. Instead, the QLB_LQL-T_ in our study delayed the first postoperative methadone administration up to a maximum of about 11 h, when ropivacaine 0.5% was used. However, the premedication protocols administered in those studies included a combination of methadone, dexmedetomidine, and ketamine or a combination of methadone and dexmedetomidine, respectively [12,13]. In addition, all dogs received meloxicam IV at the end of the surgery. In our study, we designed an anesthetic protocol to reduce possible interferences due to other drugs. In our opinion, the decision to administer only fentanyl IV in premedication and the delayed administration of carprofen in the postoperative period allowed us to assess more precisely the perioperative analgesic effect of the QLB_LQL-T_. According to this study design, a pain score higher than or equal to 5/24 was used as cutoff value for the administration of postoperative rescue methadone, despite a score of 6/24 is suggested [31]. This decision was based on the author’s intention to treat pain as soon the analgesic effect of ropivacaine started to decrease, taking in account that dogs in our study received only fentanyl in premedication. Similarly, Viscasillas et al. used a cut-off of 4/24 in their study [14].

In human medicine, hypotension secondary to bilateral QLB has been described in two case reports [22,23]. The authors speculated that this complication was related to a bilateral paravertebral extension of the QLB leading to an associated sympathetic block, resulting in a long-lasting vasodilation. In our study, we registered a reduction in MAP in the five minutes following the execution of the block in both groups, which could be due to a certain degree of sympatholysis and splanchnic vasodilation. However, this complication did not last. Surprisingly, the reduction in MAP was statistically significant only when ropivacaine 0.33% was used. Experimental studies conducted in animals have shown that ropivacaine is able to elicit a vasoconstriction response directly proportional to the concentration used [36,37,38]. The same results have also been achieved in clinical studies [39]. Since vasoconstriction decrease the systemic absorption of local anesthetic (LA), we might speculate that the vasoconstriction induced by ropivacaine was less important in group QLB_0.33%_ than in group QLB_0.5%_. Consequently, the systemic absorption of the drug may have been greater in subjects received ropivacaine 0.33%, resulting in a transitory reduction in MAP.

Intraoperative HR, SAP, MAP, *f*_R,_ FE’Sevo, did not show statistically significant differences between the three groups. Our study design aimed to reduce possible interferences due to other drugs except for the QLB_LQL-T_ on the anesthetic and analgesic plane. This finding may suggest that the QLB_LQL-T_ with ropivacaine 0.5% and 0.33% provided a similar anesthetic and analgesic plane to a systemic fentanyl-based analgesic protocol. However, although the difference was not statistically significant, dogs in groups receiving QLB_LQL-T_ suffered less cardiovascular complications than dogs in group No-QLB, as reported in the literature [7,8].

Nine out of sixteen dogs required intraoperative rescue fentanyl in groups QLB during S_1_, S_2_, and S_3_. This result supports the notion that, during a minimally invasive surgery such as LO, the insufflation of the peritoneal cavity corresponds to an important nociceptive somatic stimulation, as reported in human medicine [40]. The canine abdominal wall is innervated by the VRSN between the ninth thoracic (T9) and L3 vertebrae [11]. Garbin et al. reported that QLB_LQL-T_ performed with 0.3 mL kg^−1^ did not produce a consistent spread to the caudal thoracic VRSN, responsible for the innervation of the cranial portion of the abdominal wall [17]. However, we decided to use 0.3 mL kg^−1^ because a higher volume in group QLB 0.5% would have exceeded 3 mg kg^−1^ of ropivacaine [7]. Therefore, our results seem to confirm findings from previous cadaveric study [17]. Four out of eighteen dogs required intraoperative rescue fentanyl during S_6_ and S_7_. Ovaries in female dogs are innervated by fibers from the sympathetic trunk portion between T13 and the second lumbar (L2) vertebrae [41], consistently stained by the QLB_LQL-T_ in a previous cadaveric study [17]. However, an important limitation of the study needs to be addressed. According to this study design, a bolus of 2.5 mcg kg^−1^ of fentanyl IV was administered in presence of nociception, in 9/16 dogs during S_1_, S_2_, and S_3_. Considering the short duration of surgery in our study (35 ± 13 and 35 ± 11 in group QLB_0.5%_ and group QLB_0.33%_, respectively), and taking in account that plasma fentanyl concentrations decrease only after 20 min after IV administration [42], it is not possible to exclude that the rescue analgesia administered during the first steps of the LO mitigated the cardiovascular response during the visceral stimulation.

Based on the results of our study, QLB_LQL-T_ seemed to produce a reduction in the requirement of intraoperative rescue fentanyl, in comparison with a group receiving a systemic fentanyl-based analgesic protocol. However, the anesthetists in charge of the intraoperative monitoring were aware of the group No-QLB due to ethical issues, which could bias the results. Despite this, no statistically significant differences were found regarding the intraoperative rescue fentanyl consumption between group QLB_0.5%_ and QLB_0.33%_, suggesting that QLB_LQL-T_ performed with ropivacaine 0.5% and 0.33% provides the same intraoperative analgesic efficacy. This finding has been already reported in previous studies [32,33,35]. This information could be clinically useful, especially when an animal with comorbidities (cardiac, renal, or hepatic diseases) may equally benefit from an IFBP and from a reduction in the total amount of LA administered [32]. 

In their study, Viscasillas et al. found that only 1/10 dog receiving the QLB required intraoperative rescue analgesia during laparotomic ovariohysterectomy [14]. The results of this study greatly differ from ours. However, a different approach to the QLB was performed [19], a different LA and volume were used and, most importantly, a substantially different premedication (medetomidine 20 mcg kg^−1^ intramuscularly and meloxicam 0.2 mg kg^−1^ subcutaneously) was administered. Considering the analgesic effect and duration of the drugs used in their study [43,44], it is not possible to exclude that the difference in rescue analgesia requirement between these two studies was dependent on drugs administered in premedication, rather than the QLB technique.

The present study has several limitations which need to be addressed. First, the study was based on a small sample of animals, and dogs were recruited from the population of a single Veterinary Teaching Hospital, which may limit generalization of the results. Second, as already mentioned, the anesthetists in charge of the intraoperative monitoring were aware of the group No-QLB, leading to a possible bias: ideally, a negative control group should have received QLB_LQL-T_ with saline solution; however, this was not considered ethically feasible in our institution. Third, despite all blocks being performed by the same operator, the success of any LRA technique is operator-dependent and it is influenced by the difficulty of the technique performed. It is not possible to rule out that different results would be obtained if blocks were performed in a higher number of dogs by different anesthetists. Part of these limitations would be addressed by designing a multicentric study. Fourth, the decision to assess postoperative pain for a limited number of hours after the block execution did not allow in some cases (particularly in group QLB_0.5%_) to identify the exact moment when the first postoperative methadone administration was needed and the total consumption of postoperative methadone. However, LO is considered a day-hospital surgical procedure and postoperative hospitalization is not always essential [5]; for this reason, we limited the post-operative time monitoring. 

## 5. Conclusions

In conclusion, bilateral QLB_LQL-T_ with a volume of 0.3 mL kg^−1^ of ropivacaine prolonged the time to the first postoperative methadone administration and reduced the pain scores in female dogs undergoing LO, in comparison with dogs receiving a systemic fentanyl-based analgesic protocol. In addition, ropivacaine 0.5% provided a longer postoperative effect in comparison with ropivacaine 0.33%, despite no differences being found in the intraoperative period between the two concentrations. Results of this study also suggests that ropivacaine 0.33% produce a more important reduction in the arterial blood pressure than ropivacaine 0.5%, during the first five minutes after performing the block. However, this effect did not extend into the intraoperative period.

## Figures and Tables

**Table 1:** Demographic data, time from the block to the starting of surgery (T_B-S_) (minutes), duration of anesthesia and surgery (minutes). Results are presented as mean and standard deviation. QLB_0.5%_, quadratus lumborum block (QLB_LQL-T)_ with ropivacaine 0.5%; QLB_0.33%_, quadratus lumborum block (QLB_LQL-T)_ with ropivacaine 0.33%. No-QLB, systemic fentanyl-based analgesic protocol.

Group	Age (Months)	Weight (kg)	T_B-S_ (Minutes)	Duration Anaesthesia (Minutes)	Duration Surgery (Minutes)
QLB_0.5%_	20 ± 12	29 ± 6	29 ± 6	107 ± 17	35 ± 13
QLB_0.33%_	21 ± 13	27 ± 5	27 ± 5	98 ± 33	35 ± 11
No-QLB	24 ± 12	30 ± 4	-	101 ± 17	39 ± 10

**Table 2:** Distribution of intraoperative rescue fentanyl boluses (2.5 mcg kg^−1^), during the specific surgery steps in the two groups receiving the quadratus lumborum block (QLB_LQL-T_). QLB_0.5%_, QLB_LQL-T_ with ropivacaine 0.5%; QLB_0.33%_, QLB_LQL-T_ with ropivacaine 0.33%. S_1_, placement of the drapes and Backhaus forceps; S_2_, skin incision; S_3_, introduction of the trocars and insufflation of the abdomen; S_4_, traction of the first ovary; S_5_, cauterization of the first ovary; S_6_, traction of the second ovary; S_7_, cauterization of the second ovary; S_8_, deflation of pneumoperitoneum and extraction of the trocars; S_9_, suturing of the skin.

Group	Dog	Distribution of Intraoperative Rescue Fentanyl Boluses								
		S_1_	S_2_	S_3_	S_4_	S_5_	S_6_	S_7_	S_8_	S_9_
QLB_0.5%_	1	x								
	2		x							
	3			x						
	4			x						
	5							x		
	6							x		
	7									
	8									
QLB_0.33%_	1	x								
	2	x								
	3			x						
	4			x						
	5			x						
	6						x			
	7							x		
	8									

## Data Availability

Data supporting the reported results can be sent to anyone interested by contacting the corresponding author.
